# CRAMP deficiency leads to a pro-inflammatory phenotype and impaired phagocytosis after exposure to bacterial meningitis pathogens

**DOI:** 10.1186/s12964-017-0190-1

**Published:** 2017-09-16

**Authors:** Eugenia Kress, Julika Merres, Lea-Jessica Albrecht, Sven Hammerschmidt, Thomas Pufe, Simone C. Tauber, Lars-Ove Brandenburg

**Affiliations:** 1 Department of Anatomy and Cell Biology, RWTH Aachen University, Wendlingweg 2, 52074 Aachen, Germany; 2 Department Genetics of Microorganisms, Interfaculty Institute for Genetics and Functional Genomics, University of Greifswald, Greifswald, Germany; 3 Department of Neurology, RWTH University Hospital Aachen, Aachen, Germany

**Keywords:** Antimicrobial peptide, Cathelicidin, Cramp, Glial cell, Innate immunity, Signal transduction

## Abstract

**Background:**

Antimicrobial peptides are important components of the host defence with a broad range of functions including direct antimicrobial activity and modulation of inflammation. Lack of cathelin-related antimicrobial peptide (CRAMP) was associated with higher mortality and bacterial burden and impaired neutrophil granulocyte infiltration in a model of pneumococcal meningitis. The present study was designed to characterize the effects of CRAMP deficiency on glial response and phagocytosis after exposure to bacterial stimuli.

**Methods:**

CRAMP-knock out and wildtype glial cells were exposed to bacterial supernatants from *Streptococcus pneumoniae* and *Neisseria meningitides* or the bacterial cell wall components lipopolysaccharide and peptidoglycan. Cell viability, expression of pro- and anti-inflammatory mediators and activation of signal transduction pathways, phagocytosis rate and glial cell phenotype were investigated by means of cell viability assays, immunohistochemistry, real-time RT-PCR and Western blot.

**Results:**

CRAMP-deficiency was associated with stronger expression of pro-inflammatory and weakened expression of anti-inflammatory cytokines indicating a higher degree of glial cell activation even under resting-state conditions. Furthermore, increased translocation of nuclear factor ‘kappa-light-chain-enhancer’ of activated B-cells was observed and phagocytosis of *S. pneumoniae* was reduced in CRAMP-deficient microglia indicating impaired antimicrobial activity.

**Conclusions:**

In conclusion, the present study detected severe alterations of the glial immune response due to lack of CRAMP. The results indicate the importance of CRAMP to maintain and regulate the delicate balance between beneficial and harmful immune response in the brain.

**Electronic supplementary material:**

The online version of this article (10.1186/s12964-017-0190-1) contains supplementary material, which is available to authorized users.

## Background

The central nervous system (CNS) is protected from penetrating pathogens by the blood-brain barrier (BBB) and by the innate immune system which is responsible for the early control of infections. Microglial cells, being the resident innate immune cells, are involved in pathogen recognition and activation of the innate immune orchestra also including the recruitment of immune cells of the adaptive immune system. They sense microbes and pathogen-derived ligands invading the CNS by pattern recognition receptors such as Toll-like receptors (TLR) and phagocytic receptors [1, 2]. Once activated, microglial cells secret a broad range of pro-inflammatory cytokines stimulating the innate immune response as well as peripheral immune cells. Astrocytes, as the other member of glial cells, mediate neuroinflammation but are also required for structural support and the maintenance of the BBB. Activated astrocytes can exacerbate the inflammatory response and aggravate the tissue damage or can promote immunosuppression and tissue regeneration, depending on the kind of stimuli [3].

Activation of the CNS’s innate immune system leads to secretion of antimicrobial peptides (AMP) which have both anti-infective and immunomodulatory properties [4]. AMP can be divided into different families based upon primary and secondary structural differences, antimicrobial potential and specific effects on host cells. An important family of the AMP are the cathelicidins [5, 6]. In humans, there is only one cathelicidin called LL-37 that functions as a mediator between the innate and adaptive immune response. It is stored in granules of neutrophil granulocytes and thought to have diverse functions such as inducing chemotaxis, neutralizing endotoxin and supporting wound healing [7]. The cathelicidin of mice and rats is called cathelin-related antimicrobial peptide (CRAMP) [8] and rCRAMP [9], respectively. Own investigations revealed an increase of LL-37 and psoriasin (another AMP) in the cerebrospinal fluid (CSF) of patients suffering from bacterial meningitis [10, 11]. Bacterial infection and exposure of glial cells to bacterial supernatants and bacterial virulence factors induced an increase of rCRAMP and increased the release of pro- as well as anti-inflammatory cytokines [10, 12–14]. Additionally, CRAMP stimulation enhanced the expression of several neurotrophins including brain-derived neurotrophic factor (BDNF), glial cell-derived neurotrophic factor (GDNF) and nerve growth factor (NGF) thereby possibly exerting neuroprotective effects [15]. Mice lacking CRAMP were more susceptible to pneumococcal infection of the CNS leading to higher bacterial burden both inside the brain (cerebellum) and outside the CNS (blood and spleen) accompanied by decreased neutrophil granulocyte infiltration and higher mortality [16].

This study was designed to explore the pathophysiology and interrelationship of glial cell function in vitro in CRAMP-knock out (KO) and wildtype (WT) glia. For this purpose, glial cells were exposed to bacterial supernatants from *Streptococcus pneumoniae* (SP) and *Neisseria meningitidis* (NM) or bacterial cell wall components such as lipopolysaccharide (LPS) and peptidoglycan (PNG). Glial cell viability, expression of various pro- and anti-inflammatory cyto- and chemokines, phagocytosis rate and glial cell activation were investigated by means of cell viability assays, immunohistochemistry, real-time RT-PCR and Western blot. Furthermore, the regulation of signal transduction pathways, such nuclear factor ‘kappa-light-chain-enhancer’ of activated B-cells (NFκB) or the anti-inflammatory signal transduction pathway heme oxygenase 1 (HO-1) was examined.

## Methods

### Reagents

Bacterial supernatants were synthesized as described in detail previously [12]. In the present study, bacterial supernatants of *N. meningitidis* (NM; ATCC 13077) and *S. pneumoniae* (SP; ATCC 6303) were used, both in a dilution of 1:100. LPS and PGN were obtained from Sigma-Aldrich, Munich, Germany. Mouse CRAMP (ISRLAGLLRKGGEKIGEKLKKIGQKIKNFFQKLVPQPE) was purchased from GenicBio Limited (Shanghai, China). The peptide was dissolved in 0.9% NaCl solution as described before [17].

### Cell culture

Isolated cerebral cortices and rostral mesencephali from CRAMP deficient or WT mice (P2 to P3) were stripped of the meninges, minced and dissociated enzymatically with trypsin from bovine pancreas (Sigma-Aldrich, Taufkirchen, Germany) in phosphate-buffered saline and 50 μg/ml DNase I (Roche Molecular Biochemicals, Mannheim, Germany) for 30 min at 37 °C and crushed mechanically with Pasteur pipettes. Astrocytes were prepared according to the protocol of McCarthy and DeVellis [18], which allows the preparation of nearly pure cultures of astrocytes (> 97%) and cultivated in Dulbecco’s modified Eagle’s medium (DMEM; PAA Laboratories, Pasching, Austria) supplemented with 10% FCS. Suspended microglial cells were plated in 75 cm^2^ cell culture flasks (Sarstedt, Nümbrecht, Germany) in microglial cell growth medium and harvested as described previously [19]. The microglial cell growth medium (DMEM) contains 10% FCS (heat inactivating from 44 to 53 °C) and antibiotics (penicillin and streptomycin). After about ten days, the cells begin to move away from the cell layer and swim in the supernatant. The cells are collected and then seeded in normal medium (DMEM, 10% FCS heat inactivated at 56 °C, penicillin and streptomycin). Prior to replating microglial cells for different assays, cell number and viability were estimated by trypan blue exclusion. This procedure increased the purity of the microglial preparation to >98% with only very few remaining astrocytes.

### CellTiter-blue cell viability assay

The CellTiter-Blue (CTB) assay (Promega, Mannheim, Germany) was used to measure the viability of glial cells after bacterial exposure. The cells were seeded in 96-well plates and starved overnight by adding FCS-free medium. The cells were treated with different bacterial supernatants (NM and SP) or components (LPS and PGN) for 24 h. A CellTiter-Blue assay was used according to the manufacturer’s recommendations. Spectrophotometric evaluations were performed after 1, 2, and 4 h, respectively and exposed cells were compared with untreated control cells.

### LDH-assay

The lactate dehydrogenase (LDH) assay (Cayman, USA) was used to measure the cytotoxicity of glial cells after treatment. The cells were seeded in 96-well plates and starved overnight by adding FCS-free medium. Subsequent, the cells were treated with different bacterial supernatants (NM and SP) or components (LPS and PGN) for 24 h. The LDH cytotoxicity assay was used according to the manufacturer’s instructions. Spectrophotometric evaluations were performed after 30 min and exposed cells were compared with untreated cells.

### Phagocytosis and intracellular survival assay

The assay was performed according the protocol of Ribes et al. [20]. CRAMP-WT or CRAMP-KO microglial cells were exposed to noncapsulated *S. pneumoniae* R6 strain (with a ratio of approximately 50 bacteria per phagocyte). Phagocytosis was left to proceed for 30 or 90 min at 37 °C and 5% CO_2_. After bacterial exposure, cells were incubated for 1 h in culture medium containing gentamicin to kill the extracellular bacteria (final concentration, 200 μg/ml; Sigma-Aldrich, Munich, Germany). After gentamicin incubation, the cell monolayers were washed and lysed with distilled water for 5 min. The intracellular bacteria were enumerated by quantitative plating of serial dilutions of the lysates on sheep blood agar plates. The detection limit was 10 CFU/well. Each protocol was performed at least three times in independent experiments.

To monitor intracellular survival and replication inside microglial cells, cells were allowed to phagocytose bacteria for 30 min. Thereafter, cells were washed and incubated in culture medium containing gentamicin (200 μg/ml) for 2 h. At various times (30, 60, 90, and 120 min), the monolayers were washed and lysed with distilled water, and the amounts of intracellular viable bacteria were quantitatively determined.

### RNA isolation and real-time RT-PCR

Total RNA was isolated using the peqGold Trifast reagent (Peqlab, Erlangen, Germany) according to the manufacturer’s instructions. RNA samples were reverse-transcribed by moloney murine leukemia virus (MMLV) reverse transcriptase (Fermentas, Burlington, Canada) and random hexamer primers (Invitrogen, Darmstadt, Germany). The cDNA products were used immediately for SYBR green (Applied Biosystems, Darmstadt, Germany) real-time RT-PCR. Gene expression was monitored using the StepOne Plus apparatus (Applied Biosystems, Darmstadt, Germany) according to manufacturer’s protocol [19]. Relative quantification was performed using the ΔCt method which results in ratios between target genes and a housekeeping reference gene (18 s). All primers were manufactured by Eurofins MWG Operon (Ebersberg, Germany). The primer sequences of interleukin-6 (IL-6), tumor necrosis factor-α (TNF-α) [16], IL-1 receptor antagonist (IL-1RA), heme oxygenase 1 (HO-1) [21], Chemokine (C-C motif) ligand 2 (CCL2), CCL3 [22] and 18 s [23] were published before, whereas the sequences for caspase 3 (Casp3), arginase 1 (Arg-1), phospholipase D1 (PLD1), PLD2, receptor for advanced glycation endproducts (RAGE) and macrophage receptor with collagenous structure (MARCO) are referred in Table 1. The specificity of the amplification reaction was determined by melting curve analysis.

**Table 1:** Primer sequences for real-time RT-PCR gene analysis

Primer		Sequence	Annealing Tm [°C]
mArg-1	for rev	5′- AAGGACAGCCTCGAGGAGGGGTAG −3′ 5′- TGGACCTCTGCCACCACACCA −3’	59
mPLD1	for rev	5’- AGATTTGCCTTGAGTTTGCGG −3′ 5′- AGATGGTGGCATTGTTCTCCG −3’	58
mPLD2	for rev	5’- TTCAGCCTCTGAAAGCACACC −3′ 5′- GTAAAGTCACCATGCGTCAAGC −3’	57
mRAGE	for rev	5’-CCCTTAGCTGGCACTTAGATGG-3′ 5′-TGACCGCAGTGTAAAGAGTCCC-3’	59
mMARCO	for rev	5’-CAGTGCCCAAGAAGAGAAATGG-3′ 5′-GAACTTGAATCAGCAGCAGTGC-3’	57

### Fluorescence microscopy

Primary mice astrocytes or microglia were seeded on cover glasses. For intracellular staining, the cells were permeabilized with 0.1% Triton X in PBS for 10 min at room temperature. Slices were incubated at 4 °C with the following antibody: NFκB p65 (rabbit monoclonal; #8242, Cell Signaling, USA), HO-1 (goat polyclonal; ab13243, abcam, UK) or anti-pneumococcal IgG (kindly provided by Hammerschmidt et al. [24]). Finally, the slices were incubated with anti-rabbit Cy3 (AP132C, Millipore, Darmstadt, Germany) or anti-goat Alexa488 (A-11055, Invitrogen, Darmstadt, Germany) for 1 h at room temperature. Nuclear counter-staining was performed with Diamidino-2-phenylindole dihydrochloride DAPI (Sigma 9542, Munich, Germany). Cells were digitally photographed using a Keyence digital microscope (BZ-9000, Neu Isenburg, Germany). To evaluate the increase of fluorescence, the relative fluorescence intensity from the ratio of the corresponding fluorescence intensity of the glial cells compared to the untreated controls was calculated. For the negative controls, the primary antibodies were omitted. The analysis of fluorescence intensity was quantified using ImageJ. The area, integrated density and mean grey value were determined. To calculate the corrected total cell fluorescence (CTCF) the following formula was used: CTCF: Integrated density–area of selected cell x mean fluorescence of background readings: The ratio between CTCF (treated cells) and corresponding control was calculated and the values were referred to control (=100%).

### Statistical analysis

All real-time RT-PCR experiments were performed in duplicate. The values are expressed as mean ± SEM. For statistical comparison, two-way ANOVA test followed by Bonferroni’s multiple comparison test or Student’s t-test (analysis of phagocytosis rate) was used. A value of *p* < 0.05 was considered statistically significant. For statistical calculation, GraphPad Prism 5.0 was used (Graph Pad Software, San Diego, USA).

## Results

### Increased cell viability after bacterial supernatants exposure in CRAMP-deficient microglial cells

**Fig. 1:**
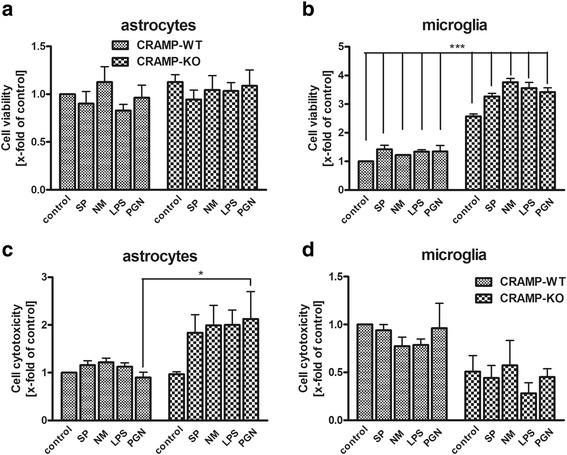
**a**-**d**. *Significant induction of metabolic activity in CRAMP-KO microglia after bacterial stimulation.* Astrocytes (**a**, **c**) and microglial cells (**b**, **d**) from CRAMP wild-type (WT) or knockout (KO) mice were incubated with bacterial supernatants of Gram-positive *S. pneumoniae* (SP) or Gram-negative *N. meningitidis* (NM) and bacterial cell wall components lipopolysaccharide (LPS) or peptidoglycan (PGN) for 24 h. Cell viability or cytotoxicity was determined using CellTiter-Blue (**a**, **b**) or lactate dehydrogenase (LDH; **c**, **d**) assay. Data were assessed from three independent experiments. An asterisk indicates a significant difference between stimulated WT and CRAMP-KO glial cells (*** - *p* < 0.001; two-way ANOVA followed by Bonferroni’s multiple comparison test)

Cell viability and cytotoxicity of primary CRAMP-deficient or WT astrocytes as well as microglial cells was determined after exposure to bacterial supernatants of SP or NM as well as the cell wall components LPS or PGN for 24 h. CRAMP deficiency significantly increased viability of microglial cells after stimulation with all agents and also the un-stimulated control (Fig. 1b). In contrast, there were no significant differences of cell viability observed in astrocytes (Fig. 1a). Cytotoxicity - assessed by measurement of LDH - revealed only for the treatment with PGN a significant increase in CRAMP-deficient astrocytes (Fig. 1c) whereas exposure to all other agents as well as stimulation of microglia did not lead to significant changes of cytotoxicity (Fig. 1d). The results reveal an increased metabolic activity of CRAMP-KO microglia and an increased vulnerability of CRAMP-KO astrocytes to PGN. In addition, we performed a double fluorescence staining for Ki67 as proliferation marker and TUNEL for apoptosis (details about this in the Additional file 1). The results did not show a difference between CRAMP-WT and CRAMP-KO glial cells for proliferation. Furthermore, the TUNEL staining showed no appreciable apoptosis after the different treatments in both genotypes (see Additional files 2 and 3).

### Increased pro-inflammatory cytokine expression in CRAMP-deficient glial cells after bacterial supernatants stimulation

**Fig. 2:**
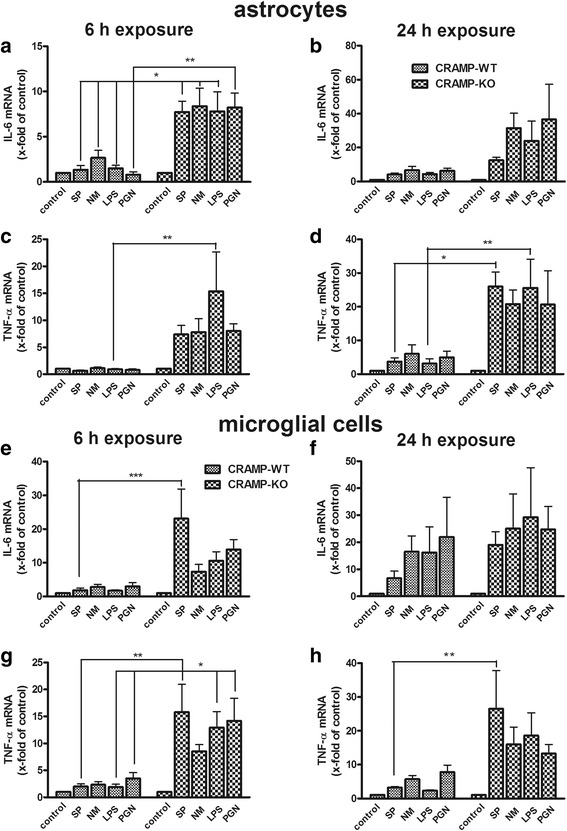
**a**-**h**. *Increased pro-inflammatory cytokine expression in CRAMP-deficient astrocytes or microglial cells after bacterial stimulation.* Astrocytes or microglial cells from CRAMP-KO or wild-type (WT) mice were incubated with bacterial supernatants of Gram-positive *S. pneumoniae* (SP) or Gram-negative *N. meningitidis* (NM) and bacterial cell wall components lipopolysaccharide (LPS) or peptidoglycan (PGN) for either 6 or 24 h. mRNA levels of Interleukin-6 (IL-6) (**a**, **b** and **e**, **f**) and Tumor necrosis factor-α (TNF-α) (**c**, **d** and **g**, **h**) were determined by real-time RT-PCR. Data were assessed from five independent experiments in duplicate. Statistical significance is marked as * - *p* < 0.05 and ** - *p* < 0.01 (two-way ANOVA test followed by Bonferroni’s multiple-comparison test)

To explore the cytokine expression pattern after 6 h and 24 h of stimulation, mRNA expression of TNF-α and IL-6 - as two major mediators of the inflammatory reaction during the innate immune response and in bacterial meningitis - were determined in astrocytes and microglial cells. In astrocytes, exposure to bacterial supernatants and components resulted in a small increase of both cytokines in CRAMP-WT, whereas in CRAMP-KO a strong increase of expression was observed (Fig. 2a and c) reaching a statistical significant increase of IL-6 after 6 h of supernatants exposure and 15.4-fold increase of TNF-α after 6 h of LPS stimulation. After 24 h of stimulation, also CRAMP-WT astrocytes showed a relevant increase of IL-6 and TNF-α mRNA expression in comparison to 6 h. However, CRAMP-KO astrocytes showed a much stronger increase of IL-6 and TNF-α expression in comparison to WT (Fig. 2b and d). For TNF-α, the differences were significant for treatment with SP and LPS (Fig. 2d). A similar observation was made in microglial cells after 6 h of stimulation with no relevant changes of cytokine expression in CRAMP-WT microglial cells whereas IL-6 and TNF-α mRNA expression was strongly increased in CRAMP-KO (Fig. 2e and g) with reaching statistical significance after SP exposure for IL-6 expression and SP, LPS or PGN for TNF-α expression. After 24 h of exposure, IL-6 mRNA expression increased in both CRAMP-WT and CRAMP-KO microglial cells without significant difference (Fig. 2f) whereas TNF-α, like in astrocytes, was strongly increased with significance after SP treatment (Fig. 2h). Altogether, the changes of cytokine expression pattern indicate a switch to a pronounced pro-inflammatory state in CRAMP-deficiency glial cells.

### Reduced expression of anti-inflammatory mediators and chemokines in CRAMP-deficient glial cells

**Fig. 3:**
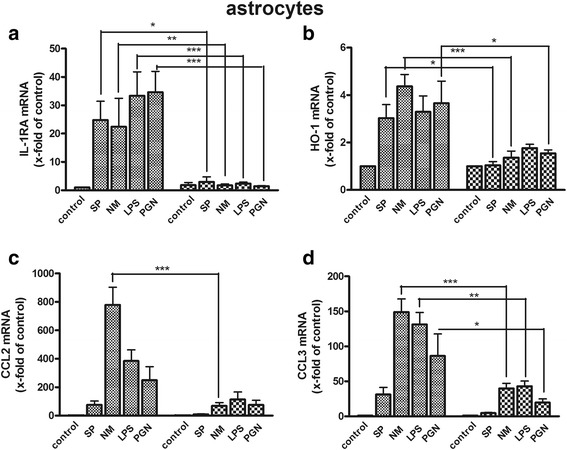
**a**-**d**. *Lower expression of anti-inflammatory mediators and chemokines in CRAMP-deficient astrocytes after bacterial stimulation.* Astrocytes from CRAMP-KO or wild-type (WT) mice were incubated with bacterial supernatants of Gram-positive *S. pneumoniae* (SP) or Gram-negative *N. meningitidis* (NM) and bacterial cell wall components lipopolysaccharide (LPS) or peptidoglycan (PGN) for 24 h. mRNA expression of Interleukin-1 receptor antagonist (IL-1RA; **a**), Haem Oxigenase 1 (HO-1; **b**), Chemokine (C-C motif) ligand 2 (CCL2; **c**) and CCL3 (**d**) was determined by real-time RT-PCR. Data were assessed from five independent experiments in duplicate. Statistical significance is marked as * - *p* < 0.05; ** - *p* < 0.01; *** - *p* < 0.001** (two-way ANOVA test followed by Bonferroni’s multiple-comparison test)

Additionally to pro-inflammatory cytokine expression, analysis of anti-inflammatory mediators and two anti-inflammatory chemokines was carried out 24 h after bacterial stimulation. IL- 1RA inhibits the pro-inflammatory response of IL-1β, which is released primarily in response to exogenous agents [25]. While IL-1RA mRNA expression was significantly increased in CRAMP-WT astrocytes by all stimulants, no relevant increase was observed in CRAMP-KO astrocytes (Fig. 3a). Additionally, the expression of the anti-inflammatory enzyme HO-1 was determined [26]. Similar to the results for IL-1RA, HO-1 expression was induced in CRAMP-WT astrocytes whereas CRAMP-KO astrocytes failed to be relevantly stimulated in HO-1 expression by bacterial stimulation (Fig. 3b). To extend the anti-inflammatory analysis, two other chemotactic mediators, CCL2 and CCL3, were added. CCL2 recruits monocytes, memory T-cells and dendritic cells to the sites of inflammation [27] and CCL3 is involved in the recruitment and activation of polymorphonuclear leukocytes [28]. Again revealing an impairment of CRAMP-KO astrocytes to release anti-inflammatory mediators, CRAMP-WT astrocytes showed a considerable increase in CCL2 and CCL3 expression while no relevant increase was observed in CRAMP-KO reaching statistical significance for CCL2 after NM exposure (Fig. 3c) and for CCL3 after exposure to NM, LPS and PGN (Fig. 3d).

**Fig. 4:**
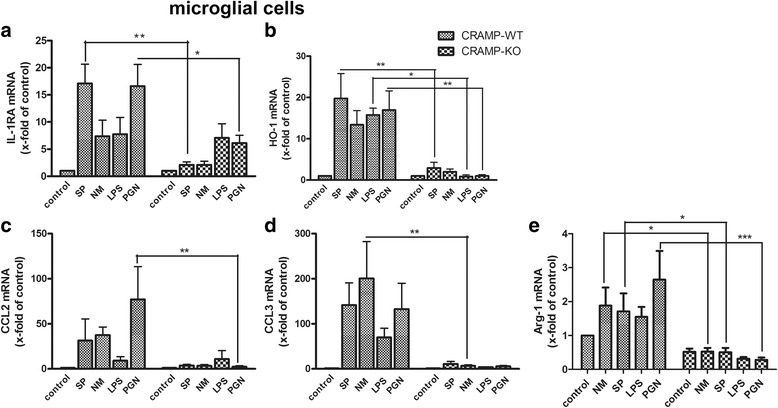
**a**-**e**. *Lower expression of anti-inflammatory mediators and marker of M2 phenotype in CRAMP-deficient microglia.* Microglial cells from CRAMP-KO or wild-type (WT) mice were incubated with bacterial supernatants of Gram-positive *S. pneumoniae* (SP) or Gram-negative *N. meningitidis* (NM) and bacterial cell wall components lipopolysaccharide (LPS) or peptidoglycan (PGN) for 24 h. mRNA expression of Interleukin-1 receptor antagonist (IL-1RA; **a**), Haem Oxigenase 1 (HO-1; **b**), Chemokine (C-C motif) ligand 2 (CCL2; **c**), CCL3 (**d**) and Arginase-1 (Arg-1; **e**) were determined by real-time RT-PCR. Data were assessed from five independent experiments in duplicate. Statistical significance is marked as * - *p* < 0.05; ** - *p* < 0.01; *** - *p* < 0.001** (two-way ANOVA test followed by Bonferroni’s multiple-comparison test)

Next, we carried out the same experiments as above with CRAMP-KO and WT microglial cells to investigate whether the observations in astrocytes were similar or even more pronounced in microglia. As shown in Fig. 4a, SP and PGN induced a strong increase of IL-1RA mRNA expression in CRAMP-WT microglial cells whereas NM and LPS had only minor effects just like all stimulants remained insufficient in inducing IL-1RA in CRAMP-KO microglia (Fig. 4a). The same tendency was shown for HO-1, where CRAMP-WT microglia responded to stimulation by increasing HO-1 expression and CRAMP-KO microglia only did insufficiently, with statistical significance for SP, LPS and PGN (Fig. 4b). Analysis of CCL2 and CCL3 expression revealed higher levels in CRAMP-WT microglial cells in comparison to CRAMP-KO. Significant differences were reached for CCL2 expression by PGN stimulation and CCL3 expression by NM exposure (Fig. 4c and d). Taken together, the results point out the imbalance between pro- and anti-inflammatory cytokine expressions with the anti-inflammatory side being impaired.

As influenced by their environment, microglial cells assume a diversity of phenotypes and retain the capability to shift functions to maintain tissue homeostasis. Therefore, next to pro- and anti-inflammatory markers, we additionally determined Arg-1 mRNA expression as a marker for alternative activation of microglial cells towards a M2 phenotype [29]. As shown in Fig. 4e, bacterial stimulation induced Arg-1 expression in CRAMP-WT microglia largely than in CRAMP-KO microglial cells (Fig. 4e). This observation confirms the changed phenotype of CRAMP-KO microglial cells.

### Comparison of signal transduction pathway activation between CRAMP-WT and CRAMP-KO glial cells after bacterial exposure

**Fig. 5:**
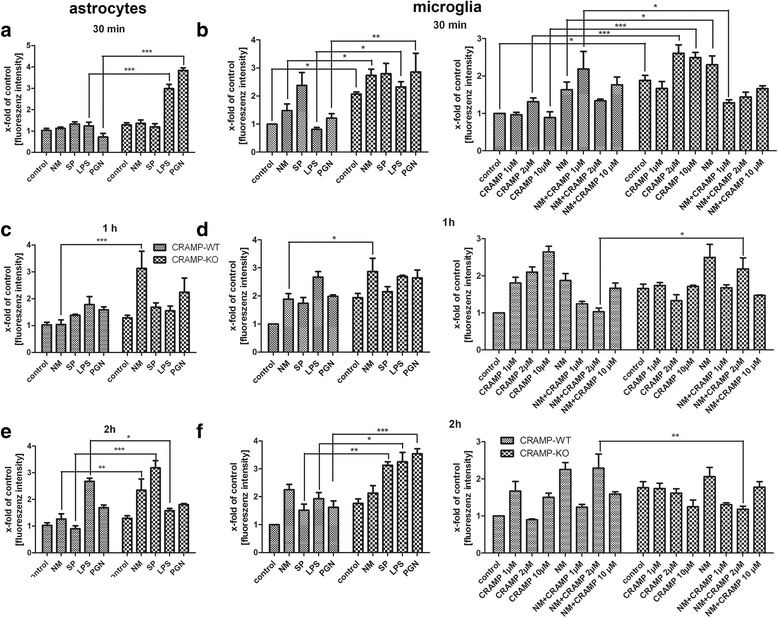
**a**-**f**. *Increased nuclear NFκB p65 translocation in CRAMP-KO glial cells after bacterial stimulation.* Astrocytes (**a**, **c**, **e**) and microglial cells (**b**, **d**, **f**, left side) from CRAMP-knockout (KO) or wild-type (WT) mice were incubated with bacterial supernatants of Gram-positive *S. pneumoniae* (SP) or Gram-negative *N. meningitidis* (NM) and bacterial cell wall components lipopolysaccharide (LPS) or peptidoglycan (PGN) for 30 min, 1 and 2 h, respectively. Microglial cells (**b**, **d**, **f**, right side) from CRAMP-KO or WT mice were incubated with 1, 2 or 10 μM mouse CRAMP with or without supernatant of NM. Fluorescence intensity was measured and calculated as the ratio of untreated control cells to stimulated cells in the cell nucleus. The experiment was performed at least in triplicate. The results represent at least three independent experiments. Statistical significance is marked as * - *p* < 0.05, ** - *p* < 0.01 or *** - *p* < 0.0001 (two-way ANOVA test followed by Bonferroni’s multiple-comparison test)

To further characterize the way leading to altered inflammatory responses between CRAMP-KO and WT glia, translocation of the pro-inflammatory transcription factor and important regulator of innate immune response, NFκB p65, was investigated by immunofluorescence detection in the nucleus of CRAMP-KO and WT astrocytes and microglia after exposure to bacterial supernatants and components. Here, the relative fluorescence intensity from the ratio of the corresponding fluorescence intensity of astrocytes or microglial cell was compared to the CRAMP-WT (for details refer to Material & Methods). Exposure to SP or NM induced only a weak NFκB translocation to the cell nucleus whereas treatment with LPS or PGN resulted in a stronger increase with a maximum after 2 h (Fig. 5a–e). In contrast, CRAMP-KO astrocytes already showed a robust increase of NFκB translocation 30 min after LPS and PGN exposure whereas the maximum increase after stimulation with NM and SP was later detected, after 1 and 2 h, respectively.

Already in resting state, CRAMP-KO microglial cells displayed significantly higher levels of NFκB translocation to the cell nucleus in comparison to un-stimulated WT microglia (Fig. 5b). And already after 30 min, CRAMP-KO microglial cells displayed significantly higher levels of NFκB immunofluorescence in the cell nucleus after exposure to NM, LPS or PGN compared to WT microglia. Maximum levels were reached in CRAMP-KO microglia for NM after 1 h and for SP, LPS and PGN after 2 h (Fig. 5d and f) revealing an altered resting state and stronger activation of this pathway in CRAMP-KO microglia.

As a try of proof of principle, we additionally analyzed whether exogenous CRAMP application could reduce the pathologically increased NFκB translocation in CRAMP-KO microglial cells. For this purpose, CRAMP-KO and WT microglial cells were exposed to bacterial supernatant of NM with or without different CRAMP concentrations (1, 2 or 10 μM) or CRAMP alone for 30 min up to 2 h. Additional file 4 showed representative results from the immunofluorescence analysis. Whereas CRAMP-WT microglial cells did not respond relevantly to additional CRAMP stimulation, CRAMP-KO microglia displayed a reduction of NFκB translocation after co-stimulation (Fig. 5b, right). However, CRAMP stimulation only (2 and 10 μM) induced a strong NFκB translocation to the cell nucleus in CRAMP-KO microglial cells after 30 min. After 1 h of treatment, stimulation with CRAMP alone induced a concentration-dependent increase of NFκB translocation whereas co-stimulation reversed this effect (Fig. 5d, right). In contrast, no CRAMP-induced increase of NFκB translocation was detected in CRAMP-KO microglial cells after 1 and 2 h of stimulation while co-stimulation resulted in a tendency of decreasing the NM-induced translocation (Fig. 5d and f, right). After 2 h of treatment of CRAMP-WT microglial cells, 1 and 10 μM CRAMP induced an increase of NFκB translocation, whereas the co-stimulation with 1 and 10 μM CRAMP reduced NM-induced translocation (Fig. 5f, right). Taken together, exogenous application of CRAMP to CRAMP-KO glial cells partly reversed the overactivated NFκB translocation, especially in combination with NM.

**Fig. 6:**
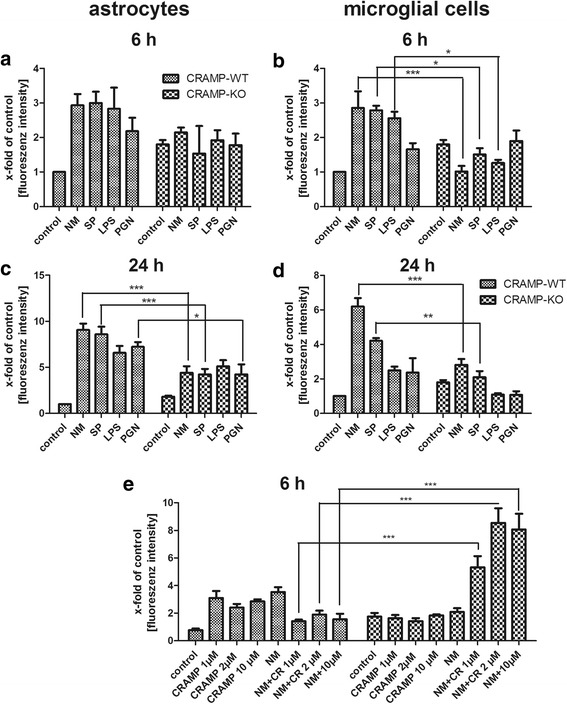
**a**-**e**. *Reduced heme oxygenase-1 induction in CRAMP-KO glial cells after bacterial stimulation.* Astrocytes (**a**, **c**) and microglial cells (**b**, **d**) from CRAMP-knockout (KO) or wild-type (WT) mice were incubated with bacterial supernatants of Gram-positive *S. pneumoniae* (SP) or Gram-negative *N. meningitidis* (NM) and bacterial cell wall components lipopolysaccharide (LPS) or peptidoglycan (PGN) for 6 or 24 h. Microglial cells (**e**) from CRAMP-KO or WT mice were incubated with 1, 2 or 10 μM mouse CRAMP with or without NM for 6 h. Fluorescence intensity of the whole cell was measured and calculated as the ratio of untreated control cells to treated cells. The experiment was independently performed at least three times. The immunofluorescence results were calculated for >20 separate cells per experiment. Statistical significance is marked as * - *p* < 0.05, ** - *p* < 0.01 or *** - *p* < 0.0001 (two-way ANOVA test followed by Bonferroni’s multiple-comparison test)

As a supplement of the above pro-inflammatory analysis, investigations on the activation of the anti-inflammatory enzyme HO-1 were added by means of immunofluorescence detection (for details please refer to Material & Methods). As shown in Fig. 6a and c, stimulation of CRAMP-WT astrocytes for 6 and 24 h induced a strong increase of HO-1 immunofluorescence by all stimulants whereas CRAMP-KO astrocytes failed to respond in a similar way and displayed only slight increases after 6 h. After 24 h, the difference for NM, SP or PGN exposure reached statistical significance in comparison to CRAMP-WT astrocytes (Fig. 6a and c). Similar results were obtained after stimulation of CRAMP-KO and WT microglial cells. Already after 6 h but also still after 24 h, CRAMP-KO microglial cells displayed significantly lower levels of HO-1 immunofluorescence density after stimulation with NM, SP and LPS (Fig. 6b and d).

Again as proof of principle, we investigated whether exogenous CRAMP application could increase HO-1 immunofluorescence density in CRAMP-KO microglial cells. Cells were exposed to bacterial supernatants from NM with or without different CRAMP concentrations (1, 2 or 10 μM) or CRAMP alone for 6 h. Additional file 5 showed representative results from the immunofluorescence analysis. CRAMP application increased HO-1 immunofluorescence density considerably in CRAMP-WT microglial cells (Fig. 6e) whereas the NM-induced increase of HO-1 immunofluorescence density was weakened by CRAMP application. In CRAMP-KO microglia, exogenous CRAMP failed to increase HO-1 immunofluorescence density whereas interestingly co-stimulation of CRAMP with NM supernatant induced a statistically highly significant increase of HO-1 immunofluorescence density compared to CRAMP-WT microglial cells (Fig. 6e).

### CRAMP-deficiency resulted in a decreased microglial phagocytosis rate of *S. pneumoniae*

**Fig. 7:**
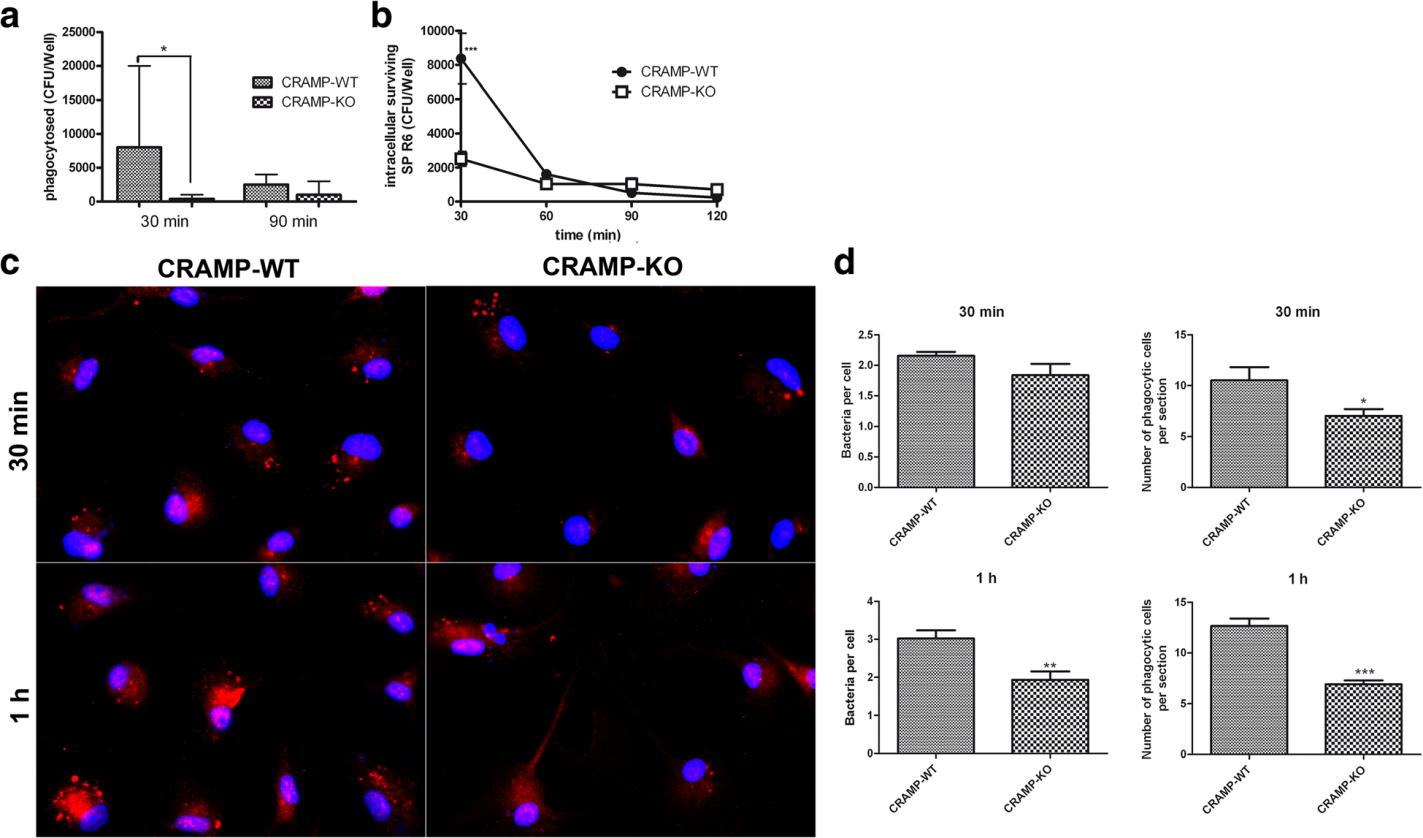
**a**-**d**. *CRAMP-deficiency resulted in decreased microglial phagocytosis rate of Streptococcus pneumoniae.* Microglial cells from CRAMP-KO or wild-type (WT) mice were exposed to *S. pneumoniae* (strain R6) for 30 and 90 min. The intracellular bacteria were enumerated by quantitative plating of the lysates on sheep blood agar plates (**a**). To evaluate intracellular survival of bacteria inside microglial cells, microglial cells from CRAMP-KO or WT were exposed to *S. pneumoniae* (strain R6) for 30 min and bacteria quantitatively dertermined at various time points (for details refer to Material & Methods) (**b**). (**c**) Microglial cells from CRAMP-KO or WT were exposed to *S. pneumoniae* (strain R6) for 30 min and 1 h. The intracellular bacteria were labelled by immunofluorescence with a rabbit anti-pneumococcal antibody and detected by anti-rabbit anti-Cy3 secondary antibody (in red; cell nucleus in blue). (**d**) Intracellular bacteria were quantified und analysed. These results were calculated for at least 20 separate cells. The experiments were performed independently at least three times. Statistical significance is marked as * - *p* < 0.05, ** - *p* < 0.01 or *** - *p* < 0.0001 (Student’s -test)

Since the present study proved altered responses of CRAMP-KO microglia to inflammatory stimuli, we extended the investigations and determined the phagocytic potential. Microglial cells were incubated with uncapsulated *S. pneumoniae* R6 strain for 30, 60, 90 and 120 min, respectively, and the amount of phagocytosed bacteria determined. The phagocytosis rate of pneumococci was compared quantitatively after 30 and 90 min. CRAMP-KO microglial cells showed a significantly lower phagocytic activity compared to WT microglia (Fig. 7a). After 90 min, no significant differences were detected. Next, we studied the intracellular surviving rate of the ingested bacteria (Fig. 7b). The absolute amounts of intracellular *S. pneumoniae* R6 were significantly higher in WT microglial cells than in CRAMP-KO microglia after 30 min of incubation. The further time course of intracellular surviving *S. pneumoniae* R6 was similar between CRAMP-KO and WT microglia.

Additionally, we highlighted intracellular *S. pneumoniae* R6 bacteria in CRAMP-KO and WT microglial cells using immunofluorescence (Fig. 7c) and quantified the intracellular bacteria after treatment for 30 min or 1 h (Fig. 7d). After 30 min, there were no significant difference in bacteria per cell between the genotypes, but the phagocytosed bacteria per section were significantly reduced in CRAMP-KO (Fig. 7d, top right). Exposure of CRAMP-KO microglial cells with bacteria for 1 h resulted in a significantly decreased amount of intracellular bacteria per cell and per section compared to WT microglia.

**Fig. 8:**
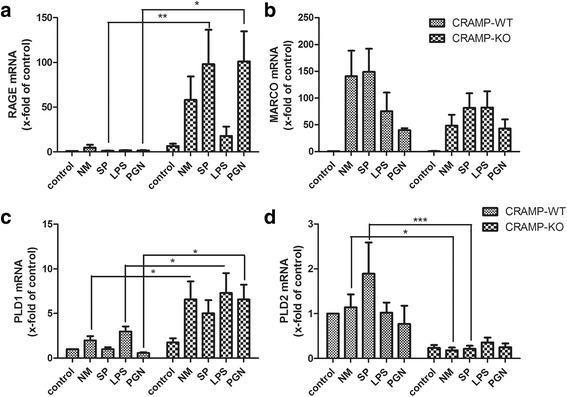
**a**-**d**. *Altered expression of phagocytosis markers in CRAMP-deficient microglial cells after bacterial stimulation.* Microglial cells from CRAMP-KO or wild-type (WT) mice were incubated with bacterial supernatants of Gram-positive *S. pneumoniae* (SP) or Gram-negative *N. meningitidis* (NM) and bacterial cell wall components lipopolysaccharide (LPS) or peptidoglycan (PGN) for 24 h. mRNA expression levels of receptor for advanced glycation endproducts (RAGE; **a**), macrophage receptor with collagenous structure (MARCO; **b**), phospholipase D1 (PLD1; **c**) and PLD2 (**d**) were determined by real-time RT-PCR. Data were assessed from five independent experiments in duplicate. Statistical significance is marked as * - *p* < 0.05, ** - *p* < 0.01 or *** - *p* < 0.0001 (two-way ANOVA test followed by Bonferroni’s multiple-comparison test)

In addition to the phagocytosis rate, we analysed different markers that are involved in the regulation of phagocytosis. RAGE elicits pro-inflammatory responses in mononuclear phagocytes [30, 31]. Analysis of RAGE mRNA expression revealed no relevant increase in CRAMP-WT microglial cells after treatment with bacterial supernatants or components for 24 h. In contrast, there was a robust increase of RAGE expression in CRAMP-KO microglia, statistically significant after exposure to SP and PGN (Fig. 8a). Next, we quantified the mRNA expression of MARCO, a receptor being involved e.g. in the defence of pneumococcal infections of the lungs [32, 33], where an increase was detected in both genotypes with a tendency of lower levels in CRAMP-KO microglia but not reaching statistical significance (Fig. 8b). At last, mRNA expression of PLD1 and PLD2 was investigated, which are both involved in phagosome maturation and processing [34]. Here, a strong increase of PDL1 was detected in CRAMP-KO microglial cells whereas the increase in WT microglia was much less pronounced (Fig. 8c). Contrarily, PLD2 expression was significantly higher in CRAMP-WT in comparison to CRAMP-KO microglia (Fig. 8d). Taken together, there were distinct changes in phagocytosis and expression rate of different markers involved in orchestrating phagocytosis.

## Discussion

In the present study, the consequences of CRAMP deficiency for glial cell function were investigated. Own previous investigations revealed that lack of CRAMP led to a higher mortality rate in bacterial meningitis; a phenomenon that was associated with increased bacterial burden and decreased neutrophil granulocyte infiltration of the meninges. CRAMP-deficient mice displayed a higher degree of glial cell activation that was accompanied by a pronounced pro-inflammatory response in a mouse model of pneumococcal meningitis [16]. The present study was designed to deepen the knowledge about the differences of the glial response in vitro after exposure to bacterial stimuli and its consequences on the phagocytosis induced by lack of CRAMP.

AMPs are participating in multiple aspects of immunity and affect various parts of the body. They are involved in septic and non-septic inflammation, wound repair, angiogenesis and regulation of the innate and adaptive immunity homeostasis [4, 35]. AMPs have distinct modulating immune functions and are able to alleviate pro-inflammatory stimuli. Incubation of human cathelicidin LL-37 significantly decreased the release of pro-inflammatory cytokines by human neutrophil granulocytes previously stimulated by LPS or bacterial supernatants [36]. Furthermore, LL-37 inhibited cellular responses to IFN-γ, a key cytokine of Th1-polarized immunity in different immune cells [37]. On the other hand, LL-37 induced glial-mediated neuroinflammation by inducing pro-inflammatory cytokine expression in human microglia [38]. Own work showed that CRAMP stimulation induced both a pro- as well as anti-inflammatory cytokine expression in glia [15]. These works illustrate the impact of CRAMP on rat glia cell function. Less is known about the consequences of CRAMP deficiency on glia cell function. The present results revealed an increase of pro-inflammatory cytokine expression exemplarily shown for TNF-α and IL-6 in CRAMP-deficient astrocytes and microglial cells in response to bacterial supernatants or bacterial cell wall components. This observation is consistent with work from Alalwani et al. who could show that neutrophil granulocytes from CRAMP-deficient mice released significantly more pro-inflammatory TNF-α after bacterial stimulation [36]. Cytokines such as TNF- α and IL-6 play an important role in bacterial meningitis to initiate and orchestrate the innate immune response in the early phase of the disease [39]. Overexpression of pro-inflammatory cytokines correlated with a stronger acute or chronic inflammation and increased glial cell density [16, 40] while lack of both cytokines resulted in a temporal and spatial disturbance of innate immune response with higher mortality, decreased glial cell activation and bacterial load after pneumococcal meningitis [41]. In contrast to the increased cytokine expression, CRAMP-deficient glial cells showed a strong decrease of the chemokines CCL2 and CCL3. CCL3 attracts polymorphonuclear leukocytes whereas CCL2 recruits monocytes, memory T-cells, and dendritic cells to the sites of inflammation [27, 28]. This decrease suggests that lack of CRAMP leads to insufficient or at least weakened chemotaxis as shown in form of reduced neutrophil granulocyte meningeal infiltration in a model of pneumococcal meningitis [16] and diminished dendritic cell trafficking into the peribronchiolar areas [42]. Increased CCL2 and CCL3 on the other hand correlated with increased neutrophil granulocyte infiltration into the CNS in infected formyl peptide receptor (FPR)-deficient mice [21]. It was suggested that CRAMP is a ligand for the G-protein coupled FPR2 [43]. Along with the increase in pro-inflammatory cytokine expression, we could show that lack of CRAMP resulted in a decreased expression of anti-inflammatory mediators such as IL-1RA and HO-1. IL-1RA is an acute phase protein which counteract the pro-inflammatory signal of IL-1 [25]. It was suggested as a therapeutic target for autoimmune diseases [44]. Due to its enzymatic activities, HO-1 exhibits anti-oxidative and anti-inflammatory properties at the same time. HO-1 metabolizes heme to billiverdin that is supposed to prevent oxidative stress and produces carbon monoxide that is able to inhibit NFκB [45, 46]. Anti-inflammatory effects of HO-1 are believed to be mediated in pulmonary infections by altered recruitment of neutrophil granulocytes from the bone marrow [47] and HO-1 might mediate neuroprotective effects in rodent stroke models [48]. Considering these data, lower levels of IL-1RA and HO-1 due to CRAMP deficiency seem to be unfavorable.

Our results suggest that lack of CRAMP shifts the balance between pro- and anti-inflammatory responses towards a more pro-inflammatory state of glial cells. Upon activation, astrocytes as well-characterized innate immune neuroglia participate in innate immune reactions and are the principal CNS source of innate inflammatory mediators [39]. Furthermore, astrocytes play a crucial role for the control and maintenance of the CNS microenvironment by influencing BBB stability and metabolic activities [49]. In response to a variety of neurologic diseases including brain trauma and infectious illnesses, astrocytes can undergo morphological and functional changes, a process known as astrogliosis [50, 51]. In the present study, CRAMP-deficient astrocytes displayed no differences concerning cell viability. Occurrence of an activated astroglial state is a phenomenon frequently observed in neuroinflammation. The compromised functional astroglial properties can at least partly be reconstituted by the anti-inflammatory dexamethasone [52] that is used as adjuvant therapy in bacterial meningitis.

CRAMP-KO microglial cells showed an increase of cell viability in comparison to CRAMP-WT whereas in contrast to astrocytes. Furthermore, microglial CRAMP-KO cells appeared to have a higher metabolic activity level already under resting state conditions. Microglial activation by diverse stimuli induces morphological changes to different unique phenotype polarization based on the classical rather pro-inflammatory M1 or alternatively the rather anti-inflammatory M2 macrophages phenotype [29, 53, 54]. The expression of the marker Arginase 1 – which favors the switch towards a M2 phenotype – was significantly decreased in CRAMP-KO microglial cells. Taken together, CRAMP-KO glial cells show a stronger inflammatory phenotype than WT glia.

Glial cell activation is linked to activation of signal transduction pathways. Our results show an activation of the pro-inflammatory signal transduction pathway NFκB in CRAMP-KO glial cells. NFκB is generally important for the regulation of the immune response [55] but little is known about the relationship of cathelicidins and NFκB. This pathway releases the NFκB subunit p65 which results in the production and secretion of pro-inflammatory cytokines such as IL-6 or TNF-α [56]. Our results show that exogenous CRAMP application in microglial cells induced also NFκB translocation in CRAMP-WT as well as CRAMP-KO microglial cells. That confirmed previous results of LL-37/CRAMP-induced glia-mediated neuroinflammation [15, 38]. For the anti-inflammatory enzyme HO-1, CRAMP-KO glial cells displayed a lower HO-1 activation after bacterial stimulation. Interestingly, exogenous CRAMP stimulation of microglial cells induced HO-1 activation in CRAMP-WT whereas CRAMP-KO showed no increase after treatment. In CRAMP-KO microglial cells, however, co-stimulation of CRAMP with NM resulted in an increase of HO-1 immunoreactivity. It can be hypothesized that the low HO-1 activity leads to lack of inhibition of NFκB with the consequence of a stronger NFκB translocation in CRAMP-KO glial cells and thereby a stronger inflammatory response in glial cells.

LL-37/CRAMP promotes bacterial phagocytosis by macrophages [57, 58]. In the present study it was shown that the phagocytic activity of CRAMP-KO microglia was significantly impaired in comparison to WT microglial cells. Similar observations were made outside the CNS in CRAMP-KO macrophages [57] as well as in CRAMP KO neutrophil granulocytes [36]. Work from Wan and colleagues indicated that LL-37 mediates antimicrobial activity by upregulating markers involved in phagocytosis such as CD14 or FcγRs [57]. We therefore investigated the expression of the scavenger receptor MARCO known to be involved in both pneumococcal and meningococcal phagocytosis by macrophages [32, 33]. However, the results suggest no major involvement of MARCO in regulating antimicrobial activity in CRAMP deficiency. By contrast, the expression of RAGE, a receptor for danger associated molecule pattern such as HMGB1 (high-mobility group box 1) and mediator of phagocytosis of apoptotic neutrophils [31, 59] was strongly increased in stimulated CRAMP-KO microglia hinting at a possible role of RAGE under these circumstances. Furthermore, RAGE elicits its pro-inflammatory responses by NF-κB translocation [31] that was increased in CRAMP-KO microglia. Additionally, we detected an inverse regulation of the PLD1 and PLD2 expression of. The both diesterases are involved in a wide range of cellular responses [60]. Whereas PLD1 was increased in stimulated CRAMP-KO microglial cells, PLD2 was decreased. PLD1 and PLD2 differ in their subcellular distributions and both enzymes are involved in different stages of phagosomes maturation and processing [34]. It is possible that the changed expression is the cause of the lack of phagocytosis in CRAMP-KO microglial cells. Altogether, lack of CRAMP resulted in significant changes of antimicrobial activity.

## Conclusions

In conclusion, the present study detected impairments of the glial immune response due to lack of the AMP CRAMP and underlines thereby the importance of CRAMP in the defense of infections within the CNS. CRAMP deficiency resulted in severe alternations of glial phenotype with switches towards a stronger pro-inflammatory and a weakened anti-inflammatory milieu and simultaneously impaired antimicrobial activity. The results indicate the impact and importance of CRAMP to maintain and regulate the delicate balance between beneficial and harmful immune response in the brain.

## Additional files

Additional file 1: Additional Material and Methods. Fluorescence microscopy of TUNEL and Ki67: Primary mice astrocytes or microglia were seeded on cover glasses. After stimulation for 24 h, the cells werr fixed with 4% paraformaldehyde. Subsequently, the cells were permeabilized with 0.1% Triton X in 0.1% sodium citrate for 3 min at 4°C. Then, the slices were incubated at 37°C for 1 h with TUNEL reaction mixture according the manufacturer’s protocol (In Situ Cell Death Detection Kit, Roche Diagnostics, Mannheim, Germany). After washing with PBS and blocking with 1.5 BSA in PBS for 10 min, the slices were incubated at 4°C about the night with Ki67 antibody (rabbit polyclonal; ab15580, abcam, UK). Finally, the slices were incubated with anti-rabbit Cy3 (AP132C, Millipore, Darmstadt, Germany) for 1 h at room temperature. Nuclear counter-staining was performed with Diamidino-2-phenylindole dihydrochloride DAPI (Sigma 9542, Munich, Germany). Cells were digitally photographed using a Keyence digital microscope (BZ-9000, Neu Isenburg, Germany). Ki67+ positive cells were counted for each treatment, where five 63×fields were evaluated. The proliferation index was determined by the number of positive cells expressing Ki67 divided by the total number of cells in each field. (DOCX 16 kb)

Additional file 2: *Proliferation and apoptosis induction after bacterial stimulation in CRAMP-WT or CRAMP-KO astrocytes.* Astrocytes from CRAMP-knockout (KO) or wild-type (WT) mice were incubated with bacterial supernatants of Gram-positive bacterium Streptococcus pneumoniae (SP) or Gram-negative bacterium Neisseria meningitidis (NM) and bacterial cell wall components lipopolysaccharide (LPS) or peptidoglycan (PGN) for 24 h. After incubation, glial cells were fixed and immunolabeled using the proliferation marker Ki67 (red), TUNEL reaction mixture for apoptosis and DAPI for nuclear counterstaining (blue). (A) Representative results from one of three independent experiments. (B) Ki67 proliferation index was calculated by the number of positive cells expressing Ki67 divided by the total number of cells in each field. These results were calculated for at least 20 separate cells. Scale bar = 20 μm. (TIFF 8603 kb)

Additional file 3: *Proliferation and apoptosis induction after bacterial stimulation in CRAMP-WT or CRAMP-KO microglial cells.* Microglial cells from CRAMP-knockout (KO) or wild-type (WT) mice were incubated with bacterial supernatants of Gram-positive bacterium Streptococcus pneumoniae (SP) or Gram-negative bacterium Neisseria meningitidis (NM) and bacterial cell wall components lipopolysaccharide (LPS) or peptidoglycan (PGN) for 24 h. After incubation, glial cells were fixed and immunolabeled using the proliferation marker Ki67 (red), TUNEL reaction mixture for apoptosis and DAPI for nuclear counterstaining (blue). (A) Representative results from one of three independent experiments. (B) Ki67 proliferation index was calculated by the number of positive cells expressing Ki67 divided by the total number of cells in each field. These results were calculated for at least 20 separate cells. Scale bar = 20 μm. (TIFF 6059 kb)

Additional file 4: *Exogenous application of CRAMP reduced NFκB translocation in CRAMP-KO microglial cells.* Microglial cells from CRAMP-WT (A) or KO (B) mice were incubated with 1, 2 or 10 μM mouse CRAMP with or without supernatant of NM for 30 min, 1 or 2 h. After incubation cells were fixed and immunolabeled using anti-NFκB p65 antibody (red) and nuclear counterstaining DAPI (blue) and examined with fluorescence microscopy. The figure shows representative results from three independent experiments. Scale bar = 20 μm. (TIFF 19383 kb)

Additional file 5: *Co-stimulation of exogenous CRAMP and bacterial supernatant NM induced increase of HO-1 immunofluorescence in CRAMP-KO microglial cells.* Microglial cells from CRAMP-WT or KO mice were incubated with 1, 2 or 10 μM mouse CRAMP with or without supernatant of NM for 6 h. After incubation cells were fixed and immunolabeled using anti-HO-1 antibody (green) and nuclear counterstaining DAPI (blue) and examined with fluorescence microscopy. The figure shows representative results from three independent experiments. Scale bar = 20 μm. (TIFF 3834 kb)

## Abbreviations

AMP: Antimicrobial peptide
CRAMP: Cathelin-related antimicrobial peptide
DMEM: Dulbecco’s modified Eagle’s medium
ERK1/2: Extracellular signal-regulated kinase 1/2
FCS: Fetal calf serum
HO-1: Heme oxygenase 1
KO: Knock out
LL-37: Human cathelicidin
NFκB: Nuclear factor ‘kappa-light-chain-enhancer’ of activated B-cells
NM: *Neisseria meningitidis*
PLD: Phospholipase D
RAGE: Receptor for advanced glycation endproducts
SP: *Streptococcus pneumoniae*
WT: Wildtype

## References

[1] Lehnardt S (2010). Innate immunity and neuroinflammation in the CNS: the role of microglia in toll-like receptor-mediated neuronal injury. Glia.

[2] Mariani MM, Kielian T (2009). Microglia in infectious diseases of the central nervous system. J NeuroImmune Pharmacol.

[3] Siffrin V, Radbruch H, Glumm R, Niesner R, Paterka M, Herz J, Leuenberger T, Lehmann SM, Luenstedt S, Rinnenthal JL (2010). In vivo imaging of partially reversible th17 cell-induced neuronal dysfunction in the course of encephalomyelitis. Immunity.

[4] Brandenburg L-O, Merres J, Albrecht L-J, Varoga D, Pufe T (2012). Antimicrobial peptides: multifunctional drugs for different applications. Polymers.

[5] Zanetti M (2005). The role of cathelicidins in the innate host defenses of mammals. Curr Issues Mol Biol.

[6] Romeo D, Skerlavaj B, Bolognesi M, Gennaro R (1988). Structure and bactericidal activity of an antibiotic dodecapeptide purified from bovine neutrophils. J Biol Chem.

[7] Kai-Larsen Y, Agerberth B (2008). The role of the multifunctional peptide LL-37 in host defense. Front Biosci.

[8] Gallo RL, Kim KJ, Bernfield M, Kozak CA, Zanetti M, Merluzzi L, Gennaro R (1997). Identification of CRAMP, a cathelin-related antimicrobial peptide expressed in the embryonic and adult mouse. J Biol Chem.

[9] Termen S, Tollin M, Olsson B, Svenberg T, Agerberth B, Gudmundsson GH (2003). Phylogeny, processing and expression of the rat cathelicidin rCRAMP: a model for innate antimicrobial peptides. Cell Mol Life Sci.

[10] Brandenburg LO, Varoga D, Nicolaeva N, Leib SL, Wilms H, Podschun R, Wruck CJ, Schroder JM, Pufe T, Lucius R (2008). Role of glial cells in the functional expression of LL-37/rat cathelin-related antimicrobial peptide in meningitis. J Neuropathol Exp Neurol.

[11] Jansen S, Podschun R, Leib SL, Grotzinger J, Oestern S, Michalek M, Pufe T, Brandenburg LO (2013). Expression and function of Psoriasin (S100A7) and Koebnerisin (S100A15) in the brain. Infect Immun.

[12] Brandenburg LO, Varoga D, Nicolaeva N, Leib SL, Podschun R, Wruck CJ, Wilms H, Lucius R, Pufe T (2009). Expression and regulation of antimicrobial peptide rCRAMP after bacterial infection in primary rat meningeal cells. J Neuroimmunol.

[13] Braun BJ, Slowik A, Leib SL, Lucius R, Varoga D, Wruck CJ, Jansen S, Podschun R, Pufe T, Brandenburg LO (2011). The formyl peptide receptor like-1 and scavenger receptor MARCO are involved in glial cell activation in bacterial meningitis. J Neuroinflammation.

[14] Redlich S, Ribes S, Schutze S, Nau R (2014). Palmitoylethanolamide stimulates phagocytosis of Escherichia Coli K1 by macrophages and increases the resistance of mice against infections. J Neuroinflammation.

[15] Brandenburg LO, Jansen S, Wruck CJ, Lucius R, Pufe T (2010). Antimicrobial peptide rCRAMP induced glial cell activation through P2Y receptor signalling pathways. Mol Immunol.

[16] Merres J, Hoss J, Albrecht LJ, Kress E, Soehnlein O, Jansen S, Pufe T, Tauber SC, Brandenburg LO (2014). Role of the cathelicidin-related antimicrobial peptide in inflammation and mortality in a mouse model of bacterial meningitis. J Innate Immun.

[17] Dorr A, Kress E, Podschun R, Pufe T, Tauber SC, Brandenburg LO (2015). Intrathecal application of the antimicrobial peptide CRAMP reduced mortality and neuroinflammation in an experimental model of pneumococcal meningitis. J Inf Secur.

[18] McCarthy KD, de Vellis J (1980). Preparation of separate astroglial and oligodendroglial cell cultures from rat cerebral tissue. J Cell Biol.

[19] Slowik A, Merres J, Elfgen A, Jansen S, Mohr F, Wruck CJ, Pufe T, Brandenburg LO (2012). Involvement of formyl peptide receptors in receptor for advanced glycation end products (RAGE) - and amyloid beta 1-42-induced signal transduction in glial cells. Mol Neurodegener.

[20] Ribes S, Ebert S, Regen T, Agarwal A, Tauber SC, Czesnik D, Spreer A, Bunkowski S, Eiffert H, Hanisch UK (2010). Toll-like receptor stimulation enhances phagocytosis and intracellular killing of nonencapsulated and encapsulated Streptococcus Pneumoniae by murine microglia. Infect Immun.

[21] Oldekamp S, Pscheidl S, Kress E, Soehnlein O, Jansen S, Pufe T, Wang JM, Tauber SC, Brandenburg LO. Lack of formyl peptide receptor 1 and 2 leads to more severe inflammation and higher mortality in mice with of pneumococcal meningitis. Immunology. 2014;143:447–461. 10.1111/imm.12324 PMC4212958 24863484

[22] Buschmann JP, Berger K, Awad H, Clarner T, Beyer C, Kipp M (2012). Inflammatory response and chemokine expression in the white matter corpus callosum and gray matter cortex region during cuprizone-induced demyelination. J Mol Neurosci.

[23] Brandenburg LO, Jansen S, Albrecht LJ, Merres J, Gerber J, Pufe T, Tauber SC (2013). CpG oligodeoxynucleotides induce the expression of the antimicrobial peptide cathelicidin in glial cells. J Neuroimmunol.

[24] Asmat TM, Agarwal V, Saleh M, Hammerschmidt S (2014). Endocytosis of Streptococcus Pneumoniae via the polymeric immunoglobulin receptor of epithelial cells relies on clathrin and caveolin dependent mechanisms. Int J Med Microbiol.

[25] Perrier S, Darakhshan F, Hajduch E (2006). IL-1 receptor antagonist in metabolic diseases: Dr Jekyll or Mr Hyde?. FEBS Lett.

[26] Cuadrado A, Rojo AI (2008). Heme oxygenase-1 as a therapeutic target in neurodegenerative diseases and brain infections. Curr Pharm Des.

[27] Yadav A, Saini V, Arora S (2010). MCP-1: chemoattractant with a role beyond immunity: a review. Clin Chim Acta.

[28] Mirabelli-Badenier M, Braunersreuther V, Viviani GL, Dallegri F, Quercioli A, Veneselli E, Mach F, Montecucco F (2011). CC and CXC chemokines are pivotal mediators of cerebral injury in ischaemic stroke. Thromb Haemost.

[29] Orihuela R, McPherson CA, Harry GJ (2016). Microglial M1/M2 polarization and metabolic states. Br J Pharmacol.

[30] Basta G, Lazzerini G, Massaro M, Simoncini T, Tanganelli P, Fu C, Kislinger T, Stern DM, Schmidt AM, De Caterina R (2002). Advanced glycation end products activate endothelium through signal-transduction receptor RAGE: a mechanism for amplification of inflammatory responses. Circulation.

[31] Han SH, Kim YH, Mook-Jung I (2011). RAGE: the beneficial and deleterious effects by diverse mechanisms of actions. Mol Cells.

[32] Arredouani M, Yang Z, Ning Y, Qin G, Soininen R, Tryggvason K, Kobzik L (2004). The scavenger receptor MARCO is required for lung defense against pneumococcal pneumonia and inhaled particles. J Exp Med.

[33] Mukhopadhyay S, Chen Y, Sankala M, Peiser L, Pikkarainen T, Kraal G, Tryggvason K, Gordon S (2006). MARCO, an innate activation marker of macrophages, is a class a scavenger receptor for Neisseria meningitidis. Eur J Immunol.

[34] Corrotte M, Chasserot-Golaz S, Huang P, Du G, Ktistakis NT, Frohman MA, Vitale N, Bader MF, Grant NJ (2006). Dynamics and function of phospholipase D and phosphatidic acid during phagocytosis. Traffic.

[35] Xhindoli D, Pacor S, Benincasa M, Scocchi M, Gennaro R, Tossi A (1858). The human cathelicidin LL-37--a pore-forming antibacterial peptide and host-cell modulator. Biochim Biophys Acta.

[36] Alalwani SM, Sierigk J, Herr C, Pinkenburg O, Gallo R, Vogelmeier C, Bals R (2010). The antimicrobial peptide LL-37 modulates the inflammatory and host defense response of human neutrophils. Eur J Immunol.

[37] Nijnik A, Pistolic J, Wyatt A, Tam S, Hancock RE (2009). Human cathelicidin peptide LL-37 modulates the effects of IFN-gamma on APCs. J Immunol.

[38] Lee M, Shi X, Barron AE, McGeer E, McGeer PL (2015). Human antimicrobial peptide LL-37 induces glial-mediated neuroinflammation. Biochem Pharmacol.

[39] Ransohoff RM, Brown MA (2012). Innate immunity in the central nervous system. J Clin Invest.

[40] Rubio-Perez JM, Morillas-Ruiz JM (2012). A review: inflammatory process in Alzheimer's disease, role of cytokines. Sci World J.

[41] Albrecht LJ, Tauber SC, Merres J, Kress E, Stope MB, Jansen S, Pufe T, Brandenburg LO (2016). Lack of Proinflammatory cytokine Interleukin-6 or tumor necrosis factor Receptor-1 results in a failure of the innate immune response after bacterial meningitis. Mediat Inflamm.

[42] Chen K, Liu M, Liu Y, Wang C, Yoshimura T, Gong W, Le Y, Tessarollo L, Wang JM (2013). Signal relay by CC chemokine receptor 2 (CCR2) and formylpeptide receptor 2 (Fpr2) in the recruitment of monocyte-derived dendritic cells in allergic airway inflammation. J Biol Chem.

[43] Kurosaka K, Chen Q, Yarovinsky F, Oppenheim JJ, Yang D (2005). Mouse cathelin-related antimicrobial peptide chemoattracts leukocytes using formyl peptide receptor-like 1/mouse formyl peptide receptor-like 2 as the receptor and acts as an immune adjuvant. J Immunol.

[44] Volarevic V, Al-Qahtani A, Arsenijevic N, Pajovic S, Lukic ML (2010). Interleukin-1 receptor antagonist (IL-1Ra) and IL-1Ra producing mesenchymal stem cells as modulators of diabetogenesis. Autoimmunity.

[45] Piantadosi CA, Withers CM, Bartz RR, MacGarvey NC, Fu P, Sweeney TE, Welty-Wolf KE, Suliman HB (2011). Heme oxygenase-1 couples activation of mitochondrial biogenesis to anti-inflammatory cytokine expression. J Biol Chem.

[46] Wei Y, Chen P, de Bruyn M, Zhang W, Bremer E, Helfrich W (2010). Carbon monoxide-releasing molecule-2 (CORM-2) attenuates acute hepatic ischemia reperfusion injury in rats. BMC Gastroenterol.

[47] Konrad FM, Braun S, Ngamsri KC, Vollmer I, Reutershan J (2014). Heme oxygenase-1 attenuates acute pulmonary inflammation by decreasing the release of segmented neutrophils from the bone marrow. Am J Physiol Lung Cell Mol Physiol.

[48] Gao Y, Xu X, Chang S, Wang Y, Xu Y, Ran S, Huang Z, Li P, Li J, Zhang L (2015). Totarol prevents neuronal injury in vitro and ameliorates brain ischemic stroke: potential roles of Akt activation and HO-1 induction. Toxicol Appl Pharmacol.

[49] Alvarez JI, Katayama T, Prat A (2013). Glial influence on the blood brain barrier. Glia.

[50] Sofroniew MV, Vinters HV (2010). Astrocytes: biology and pathology. Acta Neuropathol.

[51] Tauber SC, Ribes S, Ebert S, Heinz T, Fingerle V, Bunkowski S, Kugelstadt D, Spreer A, Jahn O, Eiffert H, Nau R (2011). Long-term intrathecal infusion of outer surface protein C from Borrelia burgdorferi causes axonal damage. J Neuropathol Exp Neurol.

[52] Hinkerohe D, Smikalla D, Schoebel A, Haghikia A, Zoidl G, Haase CG, Schlegel U, Faustmann PM (2010). Dexamethasone prevents LPS-induced microglial activation and astroglial impairment in an experimental bacterial meningitis co-culture model. Brain Res.

[53] Kettenmann H, Hanisch UK, Noda M, Verkhratsky A (2011). Physiology of microglia. Physiol Rev.

[54] Melief J, Koning N, Schuurman KG, Van De Garde MD, Smolders J, Hoek RM, Van Eijk M, Hamann J, Huitinga I (2012). Phenotyping primary human microglia: tight regulation of LPS responsiveness. Glia.

[55] Rivest S (2009). Regulation of innate immune responses in the brain. Nat Rev Immunol.

[56] Li Q, Verma IM (2002). NF-kappaB regulation in the immune system. Nat Rev Immunol.

[57] Wan M, van der Does AM, Tang X, Lindbom L, Agerberth B, Haeggstrom JZ (2014). Antimicrobial peptide LL-37 promotes bacterial phagocytosis by human macrophages. J Leukoc Biol.

[58] Zhang X, Bajic G, Andersen GR, Christiansen SH, Vorup-Jensen T (1864). The cationic peptide LL-37 binds mac-1 (CD11b/CD18) with a low dissociation rate and promotes phagocytosis. Biochim Biophys Acta.

[59] Friggeri A, Banerjee S, Biswas S, de Freitas A, Liu G, Bierhaus A, Abraham E (2011). Participation of the receptor for advanced glycation end products in efferocytosis. J Immunol.

[60] Brandenburg LO, Pufe T, Koch T (2014). Role of phospholipase d in g-protein coupled receptor function. Membranes (Basel).

