# Supplementary material for: CRAMP deficiency leads to a pro-inflammatory phenotype and impaired phagocytosis after exposure to bacterial meningitis pathogens
Source: Cell Commun Signal. 2017 Sep 16;15:32. doi: 10.1186/s12964-017-0190-1 (PMC5602852; doi:10.1186/s12964-017-0190-1)
Supplement: Supplementary file 1 — Additional Material and Methods. Fluorescence microscopy of TUNEL and Ki67: Primary mice astrocytes or microglia were seeded on cover glasses. After stimulation for 24 h, the cells werr fixed with 4% paraformaldehyde. Subsequently, the cells were permeabilized with 0.1% Triton X in 0.1% sodium citrate for 3 min at 4°C. Then, the slices were incubated at 37°C for 1 h with TUNEL reaction mixture according the manufacturer’s protocol (In Situ Cell Death Detection Kit, Roche Diagnostics, Mannheim, Germany). After washing with PBS and blocking with 1.5 BSA in PBS for 10 min, the slices were incubated at 4°C about the night with Ki67 antibody (rabbit polyclonal; ab15580, abcam, UK). Finally, the slices were incubated with anti-rabbit Cy3 (AP132C, Millipore, Darmstadt, Germany) for 1 h at room temperature. Nuclear counter-staining was performed with Diamidino-2-phenylindole dihydrochloride DAPI (Sigma 9542, Munich, Germany). Cells were digitally photographed using a Keyence digital microscope (BZ-9000, Neu Isenburg, Germany). Ki67+ positive cells were counted for each treatment, where five 63×fields were evaluated. The proliferation index was determined by the number of positive cells expressing Ki67 divided by the total number of cells in each field. (DOCX 16 kb) [file 12964_2017_190_MOESM1_ESM.docx]

**Additional Material and Methods**

*Fluorescence microscopy of TUNEL and Ki67:* Primary mice astrocytes or microglia were seeded on cover glasses. After stimulation for 24 h, the cells werr fixed with 4% paraformaldehyde. Subsequently, the cells were permeabilized with 0.1% Triton X in 0.1% sodium citrate for 3 min at 4°C. Then, the slices were incubated at 37°C for 1 h with TUNEL reaction mixture according the manufacturer’s protocol (In Situ Cell Death Detection Kit, Roche Diagnostics, Mannheim, Germany). After washing with PBS and blocking with 1.5 BSA in PBS for 10 min, the slices were incubated at 4°C about the night with Ki67 antibody (rabbit polyclonal; ab15580, abcam, UK). Finally, the slices were incubated with anti-rabbit Cy3 (AP132C, Millipore, Darmstadt, Germany) for 1 h at room temperature. Nuclear counter-staining was performed with Diamidino-2-phenylindole dihydrochloride DAPI (Sigma 9542, Munich, Germany). Cells were digitally photographed using a Keyence digital microscope (BZ-9000, Neu Isenburg, Germany). Ki67+ positive cells were counted for each treatment, where five 63×fields were evaluated. The proliferation index was determined by the number of positive cells expressing Ki67 divided by the total number of cells in each field.
