# Supplementary figures and images for: CRAMP deficiency leads to a pro-inflammatory phenotype and impaired phagocytosis after exposure to bacterial meningitis pathogens
Source: Cell Commun Signal. 2017 Sep 16;15:32. doi: 10.1186/s12964-017-0190-1 (PMC5602852; doi:10.1186/s12964-017-0190-1)

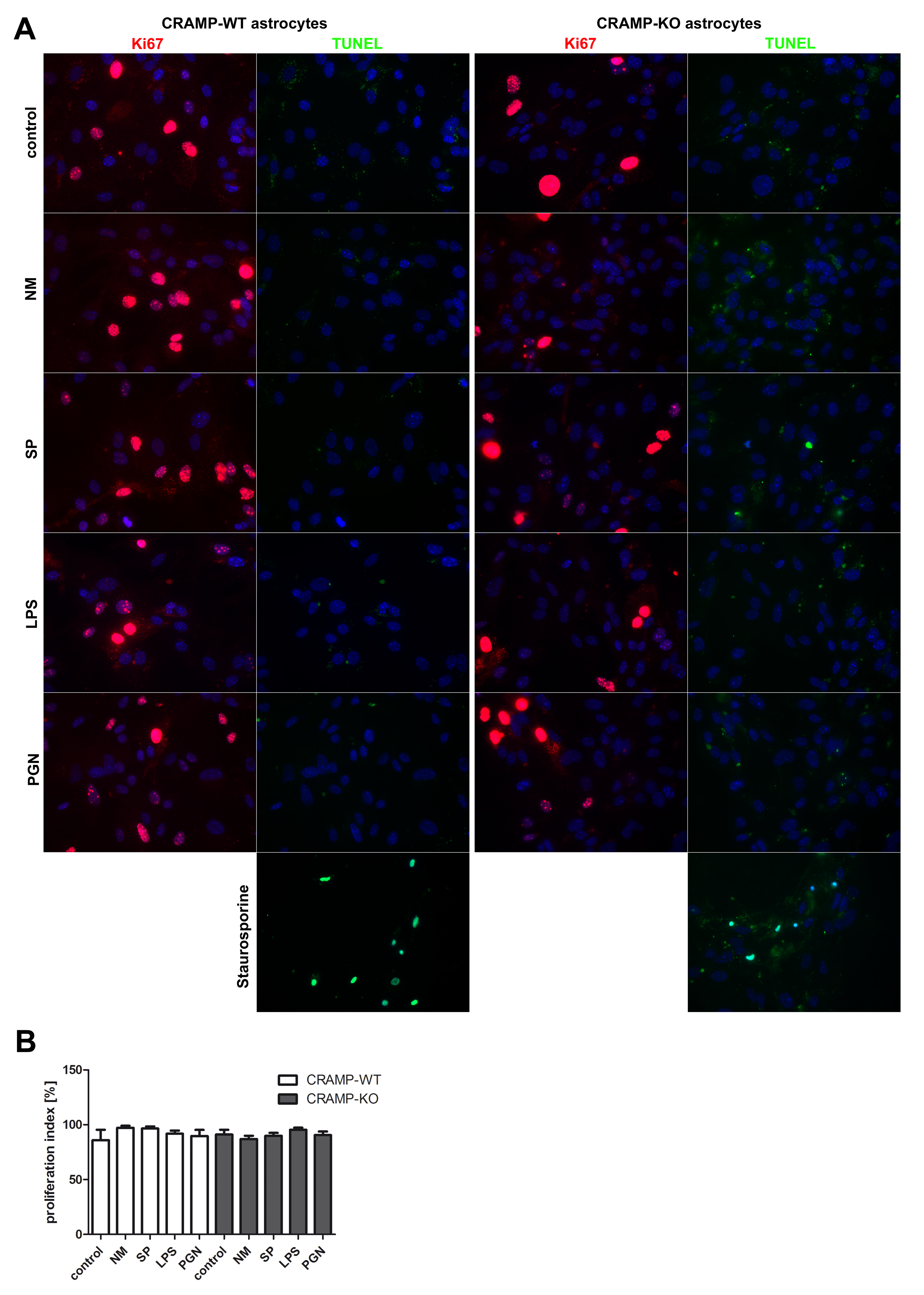

Supplement: Supplementary file 2 — Proliferation and apoptosis induction after bacterial stimulation in CRAMP-WT or CRAMP-KO astrocytes. Astrocytes from CRAMP-knockout (KO) or wild-type (WT) mice were incubated with bacterial supernatants of Gram-positive bacterium Streptococcus pneumoniae (SP) or Gram-negative bacterium Neisseria meningitidis (NM) and bacterial cell wall components lipopolysaccharide (LPS) or peptidoglycan (PGN) for 24 h. After incubation, glial cells were fixed and immunolabeled using the proliferation marker Ki67 (red), TUNEL reaction mixture for apoptosis and DAPI for nuclear counterstaining (blue). (A) Representative results from one of three independent experiments. (B) Ki67 proliferation index was calculated by the number of positive cells expressing Ki67 divided by the total number of cells in each field. These results were calculated for at least 20 separate cells. Scale bar = 20 μm. (TIFF 8603 kb) [file 12964_2017_190_MOESM2_ESM.tif]

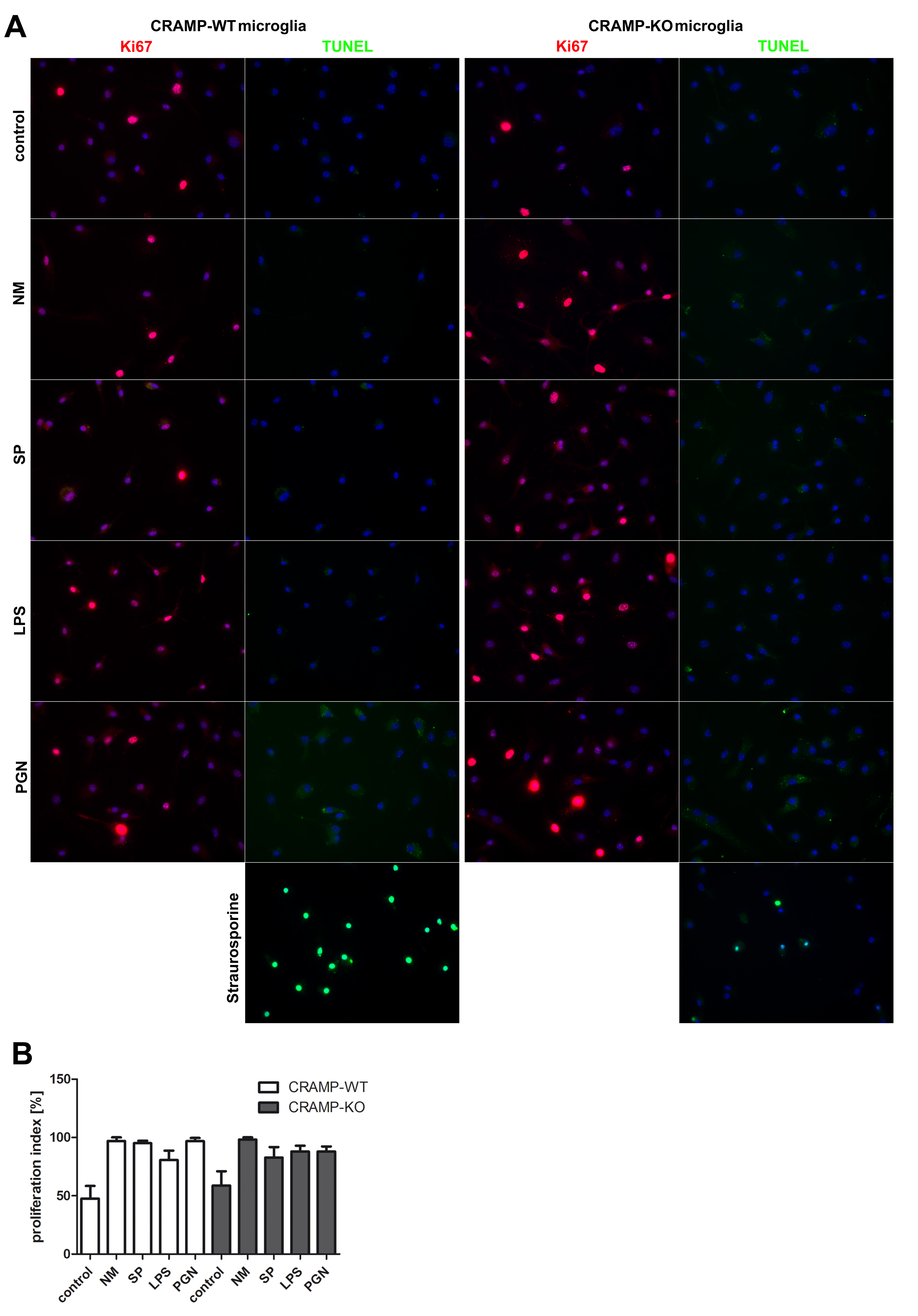

Supplement: Supplementary file 3 — Proliferation and apoptosis induction after bacterial stimulation in CRAMP-WT or CRAMP-KO microglial cells. Microglial cells from CRAMP-knockout (KO) or wild-type (WT) mice were incubated with bacterial supernatants of Gram-positive bacterium Streptococcus pneumoniae (SP) or Gram-negative bacterium Neisseria meningitidis (NM) and bacterial cell wall components lipopolysaccharide (LPS) or peptidoglycan (PGN) for 24 h. After incubation, glial cells were fixed and immunolabeled using the proliferation marker Ki67 (red), TUNEL reaction mixture for apoptosis and DAPI for nuclear counterstaining (blue). (A) Representative results from one of three independent experiments. (B) Ki67 proliferation index was calculated by the number of positive cells expressing Ki67 divided by the total number of cells in each field. These results were calculated for at least 20 separate cells. Scale bar = 20 μm. (TIFF 6059 kb) [file 12964_2017_190_MOESM3_ESM.tif]

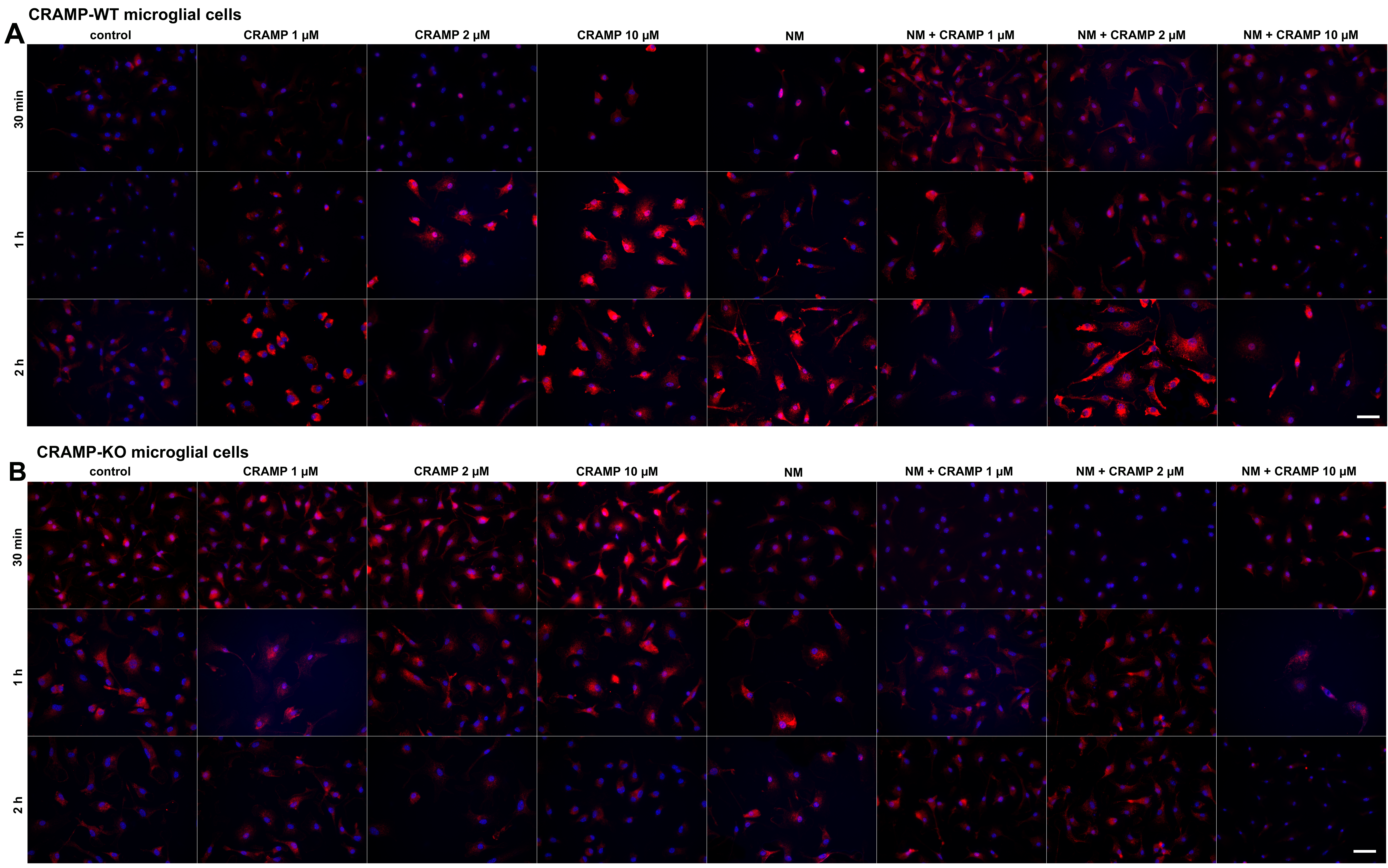

Supplement: Supplementary file 4 — Exogenous application of CRAMP reduced NFκB translocation in CRAMP-KO microglial cells. Microglial cells from CRAMP-WT (A) or KO (B) mice were incubated with 1, 2 or 10 μM mouse CRAMP with or without supernatant of NM for 30 min, 1 or 2 h. After incubation cells were fixed and immunolabeled using anti-NFκB p65 antibody (red) and nuclear counterstaining DAPI (blue) and examined with fluorescence microscopy. The figure shows representative results from three independent experiments. Scale bar = 20 μm. (TIFF 19383 kb) [file 12964_2017_190_MOESM4_ESM.tif]

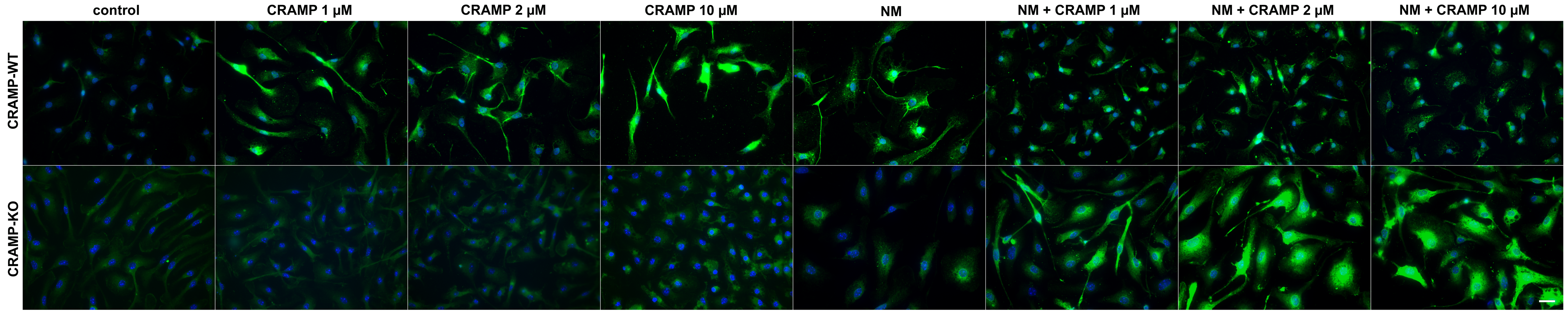

Supplement: Supplementary file 5 — Co-stimulation of exogenous CRAMP and bacterial supernatant NM induced increase of HO-1 immunofluorescence in CRAMP-KO microglial cells. Microglial cells from CRAMP-WT or KO mice were incubated with 1, 2 or 10 μM mouse CRAMP with or without supernatant of NM for 6 h. After incubation cells were fixed and immunolabeled using anti-HO-1 antibody (green) and nuclear counterstaining DAPI (blue) and examined with fluorescence microscopy. The figure shows representative results from three independent experiments. Scale bar = 20 μm. (TIFF 3834 kb) [file 12964_2017_190_MOESM5_ESM.tif]
